# Mechanistic role of lipid metabolism in foot-and-mouth disease virus (FMDV) replication

**DOI:** 10.1186/s13567-026-01762-6

**Published:** 2026-05-09

**Authors:** Yu Zhang, Fanhua Meng, Lu Li, Guoping Liu, Qi Tian, Yiyi Liu, Tao Li, Langyu Gu, Jie Gong, Chunxia Liu, Xin Wen, Fang Wan, Hongmei Xiao, Yingchun Liu, Shenyuan Wang, Junwei Cao

**Affiliations:** 1 College of Life Sciences, Inner Mongolia Agricultural University, Hohhot, 010018 China; 2 College of Agriculture, Animal Husbandry and Food, College of Hetao, Bayannur, 015000 China; 3 Inner Mongolia Key Laboratory of Biomanufacturing Technology, Hohhot, 010018 China; 4 Inner Mongolia Endemic Livestock Biotechnology Innovation Team, Hohhot, 010018 China; 5 Inner Mongolia Bayannur City Animal Husbandry Service Center, Linhe, 015000 China

**Keywords:** Foot-and-mouth disease virus, viral replication, fatty acid de novo synthesis, lipid droplets, fatty acid beta-oxidation

## Abstract

Foot-and-mouth disease (FMD) is a highly contagious animal disease caused by foot-and-mouth disease virus (FMDV), primarily affecting cloven-hoofed animals such as swine, cattle, and sheep. As a core metabolic pathway for maintaining cellular homeostasis, lipid metabolism is frequently hijacked by viruses via metabolic reprogramming mechanisms to support their infection cycle. Studies have demonstrated that positive-sense single-stranded RNA viruses can reshape the host cell membrane system and modulate the lipid metabolic network, thereby constructing a favorable microenvironment for their invasion and replication. However, the molecular mechanisms by which FMDV—also a positive-sense single-stranded RNA virus—promotes viral replication through the regulation of lipid metabolism remain incompletely elucidated. In this study, we found that inhibiting the key enzymes involved in the lipid metabolic pathway could significantly suppress FMDV proliferation. Exogenous supplementation of the downstream products catalyzed by these key enzymes notably restored FMDV replication, indicating that FMDV replication is dependent on lipids. Furthermore, we observed a significant upregulation in the protein expression of carnitine palmitoyltransferase 1A (CPT1A) in host cells following FMDV infection. Inhibition of this enzyme led to a marked reduction in FMDV replication, suggesting that FMDV may enhance the fatty acid β-oxidation pathway to supply energy for its replication. In conclusion, this study comprehensively verified the critical role of lipid metabolism in FMDV replication through multidimensional assays involving the administration of inhibitors targeting key enzymes in the lipid metabolic pathway. These findings provide novel theoretical insights for the development of antiviral drugs and the prevention and control of FMD.

## Introduction

Foot-and-mouth disease (FMD) is an acute and highly contagious animal disease caused by foot-and-mouth disease virus (FMDV), which primarily infects cloven-hoofed animals such as swine, cattle, and sheep [1]. Although this virus is generally nonfatal in adult animals, it exhibits a high mortality rate in young animals. Once an outbreak occurs, it will result in enormous economic losses [2]; therefore, FMDV has been classified as a list A animal disease by the World Organization for Animal Health (WOAH). FMDV is a positive-sense single-stranded RNA virus belonging to the genus *Aphthovirus* within the family *Picornaviridae *[3, 4]. Owing to the high cost of implementing control measures and the consequent restrictions on the export of animal products, FMDV outbreaks can inflict devastating impacts on the agriculture and animal husbandry economies. Currently, vaccination remains the primary preventive and control measure for FMDV. While vaccines play a crucial role in FMD prevention and control, they suffer from limitations, including lack of cross-protection between different serotypes, limited duration of immune protection, and high production costs. Thus, there is an urgent need to break through traditional prevention and control methods and explore novel anti-FMDV drugs or alternative prevention and control strategies.

Recent studies have demonstrated that infection of host cells by a diverse range of RNA viruses induces alterations in host cellular lipid metabolism, including enhanced de novo fatty acid synthesis, marked accumulation or depletion of lipid droplets (LDs), and accelerated fatty acid transport [5]. Positive-sense single-stranded RNA viruses such as West Nile virus (WNV) [6], hepatitis C virus (HCV) [7], and human immunodeficiency virus (HIV) [8] hijack host lipid metabolic pathways to reshape the host cell membrane system, thereby establishing a favorable microenvironment for viral invasion and replication. As is well known, fatty acids are essential structural components of cell membranes. The de novo fatty acid biosynthetic pathway involves a set of key enzymes, including acetyl coenzyme A (acetyl-CoA) carboxylase (ACC), fatty acid synthase (FASN), diacylglycerol acyltransferase 1 (DGAT-1), and CPT1A. Under the catalysis of these key enzymes, the de novo fatty acid biosynthetic pathway produces the long-chain fatty acids palmitate, triglycerides, and LDs. Among these products, triglycerides are stored in LDs and play critical roles in phospholipid synthesis, energy production, cell membrane biogenesis, and signal transduction [9]. Studies on enterovirus A71 (EV-A71) [10], classical swine fever virus (CSFV) [11], and dengue virus (DENV) [12] have revealed that inhibition of ACC and FASN—the key enzymes in de novo fatty acid synthesis—leads to a significant reduction in viral replication, indicating a close correlation between lipid metabolism and the replication of these viruses. LDs serve as central hubs for intracellular energy storage and lipid metabolism. Some viruses can induce the breakdown of LDs to release abundant free fatty acids (FFAs), which are then transported to mitochondria for β-oxidation and subsequent ATP generation. This process is termed lipophagy [13]. For instance, DENV infection promotes lipophagy to accelerate the rate of fatty acid β-oxidation, thus producing ATP to fuel viral replication [14]. During Zika virus (ZIKV) infection, the virus hijacks LDs to obtain both energy and lipid sources essential for viral replication [15]. EV-A71 upregulates the expression of carnitine CPT1A to enhance the transport efficiency of fatty acids into mitochondria, thereby supplying ATP for its replication [10]. Furthermore, emerging evidence has shown that overexpression of the FMDV 2C protein results in its targeting to the surface of LDs and induces the aggregation of LDs in the perinuclear region [16], which suggests that LDs may be involved in the replication cycle of FMDV.

Upon invading host cells, FMDV binds to receptors on the host cell surface and subsequently enters the cells via receptor-mediated endocytosis, a process that requires the support of clathrin and lipids. In FMDV-infected cells, the endoplasmic reticulum membranes undergo remodeling, and numerous novel vesicular structures emerge in the cytoplasm. These vesicles contain FMDV nonstructural proteins and host cellular factors, and they are essential for the biological processes underlying FMDV replication [17, 18]. The biogenesis of these vesicles demands substantial amounts of fatty acids, which indicates a close correlation between the host cellular lipid metabolic pathways and FMDV replication. Based on these observations, it has been well established that FMDV infection is dependent on lipids, yet the precise mechanisms by which FMDV manipulates host lipid metabolism remain elusive.

In this study, we investigated the role of lipid metabolic pathways in FMDV replication and their underlying mechanisms in depth. Our results demonstrate that inhibition of the key enzymes (ACC and FASN) in the de novo fatty acid synthesis pathway, which restricts the production of de novo fatty acids, significantly suppressed FMDV replication. Exogenous supplementation of their downstream products (malonyl-CoA and palmitic acid) remarkably restored FMDV replication. We also found that inhibition of DGAT-1, a key enzyme involved in lipid droplet biogenesis, similarly significantly inhibited FMDV replication. The addition of oleic acid (OA), which induces LD formation, restored FMDV replication, confirming the importance of lipid droplet biogenesis for FMDV replication. Furthermore, we observed that inhibition of CPT1A, a key enzyme in fatty acid β-oxidation, significantly suppressed FMDV replication, indicating the importance of the fatty acid β-oxidation pathway for FMDV replication. In conclusion, the key enzymes in de novo fatty acid synthesis (ACC and FASN) as well as other key enzymes in lipid metabolism (DGAT-1 and CPT1A) are important host factors for FMDV replication, and lipid metabolism plays a crucial role in FMDV replication.

## Materials and methods

### Cells, viruses, and reagents

The FMDV type O strain used in this study was obtained from Bureau Veritas Biotechnology Co., Ltd. in Hohhot, Inner Mongolia, and all experiments involving FMDV were performed in a biosafety level III laboratory. The hamster kidney fibroblasts (BHK-21) used in the experiments were purchased from Wuhan Punosai Life Science and Technology Co.

Acetyl-CoA carboxylase inhibitor TOFA (HY-100568) was purchased from MedChemExpress (MCE); fatty acid synthase inhibitor C75 (HY-12364) was purchased from MCE; malonyl-CoA (HY-115899) was purchased from MCE; palmitic acid (HY-N0830) was purchased from MCE; DGAT-1 inhibitor A922500 (HY-10038) was purchased from MCE; oleic acid (HY-N1446) was purchased from MCE; CPT1A inhibitors Etomoxir (HY-50202) and CP640186 (HY-15259) were purchased from MCE; and rabbit polyclonal antibody against FMDV VP1 protein (type O) (bs-41049R) was purchased from Beijing Biosynthesis Biotechnology Co., Ltd. (bioss).

### FMDV infection and amplification

BHK-21 cells seeded in T75 flasks were cultured to 70–80% confluency, followed by inoculation with 1 mL of FMDV stock solution. After 1–2 h of viral adsorption, the inoculum was discarded, and 15 mL of viral maintenance medium (low-serum MEM) was added for further incubation. When 70–80% of the cells exhibited evident cytopathic effect (CPE) under microscopy, the flask opening was sealed, and the cultures were snap-frozen at −80 ℃ for 30 min before being thawed in a 37 ℃ water bath. This freeze–thaw cycle was repeated three times to lyse the cells. The cell suspension was centrifuged, and the resulting supernatant was aliquoted into 1.5 mL sterile centrifuge tubes and stored at −80 ℃ for long-term use.

### Viral plaque assay

BHK-21 cells were seeded in 6-well plates and cultured to 60–70% confluency. Subsequently, 500 μL of diluted viral solution was added to each well, followed by incubation at 37 ℃ for 1–2 h for viral adsorption, after which the inoculum was discarded. A 1.6% agarose solution melted at 56 ℃ was mixed with prewarmed 2× Dulbecco’s modified Eagle medium (DMEM) at 37 °C at a ratio of 1:1 (yielding a final concentration of 0.8%). After cooling the mixture to 40 °C, 2 mL of the mixture was added to each well to overlay the cell monolayer. The plates were left undisturbed in a laminar flow hood for 15 min to allow the agarose to solidify, then transferred to a cell incubator for further culture at 37 °C. When visible plaques were observed by the naked eye, 1 mL of 4% paraformaldehyde was added to each well for fixation for 4 h or overnight. The agarose overlay was then removed, and the cell monolayers were stained with crystal violet for 20 min. After rinsing with phosphate-buffered saline (PBS), the number of plaques was counted to calculate the viral titer.

### CCK-8 cell viability assay

BHK-21 cells were seeded in 96-well plates and cultured to 60–70% confluency. The culture medium was then replaced, and the cells were divided into. Three groups: an inhibitor-treated group (with drug administration), a control group (without drug administration), and a blank control group (containing no cells but only cell culture medium), with eight replicate wells for each group. After 12 h of treatment, the cells were washed with PBS. Subsequently, 100 μL of minimal essential medium (MEM) and 10 μL of CCK-8 solution were added to each well, followed by incubation at 37 °C in the dark for 1–2 h. During the incubation period, the optical density (OD) values at 450 nm were measured using a microplate reader at 0.5 h, 1 h, and 2 h intervals. The time point with an OD value ranging from 0.8 to 1.2 was selected for final data recording.

### Primer design

The National Center for Biotechnology Information (NCBI) Primer-BLAST tool was used to design primers specific for the conserved regions of the target genes, and after the design was completed, primer synthesis was carried out at Kingsley Bioscience and Technology Co. The primer sequences and annealing temperatures are presented in Table 1 below.

**Table 1:** **Primer sequences and annealing temperatures**

Gene name	Species	Primer sequence	Fragment size (base pairs [bp])	Annealing temperature (℃)
FMDV-VP1	Golden hamster	F: TGTGACCAATGTGAGGGGTG R: TGGCACCGTAGTTGAAGGAG	141	59.9 60.6
β-actin	Golden hamster	F: CTCCCTCATGCCATCCTGCG R: GGCTGTGGTGGTGAAGCTGT	112	60.1 59.9
CPT1A	Golden hamster	F: TCTTCCGAGAGGGTAGGACA R: CCTCTGCTCCATCGTTTTCG	113	58.2 59.4

### Cellular RNA extraction and reverse transcription

When FMDV-infected cells in T25 flasks exhibited 50% CPE, the culture medium was discarded, and the cells were washed twice with PBS. Subsequently, 1 mL of Trizol reagent was added, and the mixture was incubated at room temperature for 5 min before being transferred to a centrifuge tube. Then, 200 μL of chloroform was added, and the tube was vortexed for 30 s, followed by incubation at room temperature for 5 min. The sample was centrifuged at 12,000 rpm at 4 °C for 15 min. The upper aqueous phase was carefully collected, mixed with an equal volume of isopropanol, and incubated at room temperature for 10 min. After centrifugation at 12,000 rpm at 4 °C for 10 min, the supernatant was discarded. The resulting RNA pellet was washed with 1 mL of prechilled 75% ethanol, followed by centrifugation at 12,000 rpm at 4 °C for 5 min. The ethanol was discarded, and a brief centrifugation was performed to collect residual ethanol, which was then aspirated completely. The RNA pellet was air-dried at room temperature for 5–10 min, then dissolved in 20 μL of RNase-free water. The purity of RNA was determined using a NanoDrop spectrophotometer (A260/A280 ratio = 1.8–2.0), and the RNA samples were stored at −80 °C. A 20 μL reverse transcription (RT) reaction system was prepared according to the Takara RR036A kit instructions, with the following thermal cycling program: 37 °C for 15 min → 85 °C for 5 s → hold at 4 °C. The resulting complementary (cDNA) was stored at −20 °C for subsequent use.

### Fluorescence-based quantitative polymerase chain reaction (PCR)

Using cDNA as the template, the reaction system was prepared with the Takara quantitative real-time PCR (qRT–PCR) kit (RR430A). After thorough mixing, the samples were subjected to qRT–PCR using a Roche LightCycler 480 II instrument.

### Western blot

When the cells reached 70–80% confluency, 250 μL of radioimmunoprecipitation assay (RIPA) lysis buffer (containing 1% PMSF) was added for cell lysis. The lysate was collected and centrifuged at 12,000 rpm at 4 °C for 20 min, and the supernatant was harvested. After determining the protein concentration using a BCA protein assay kit, the samples were stored at −80 °C. An 8% separating gel and a 5% stacking gel were prepared. Protein samples were mixed with 5× loading buffer at a ratio of 4:1, followed by denaturation at 95 °C for 5 min. A total of 20 μg of protein per well was loaded onto the gel. Electrophoresis was performed at 80 V for 1 h, then adjusted to 120 V until bromophenol blue reached the bottom of the gel. The target protein bands were excised. A PVDF membrane was activated with methanol for 1 min, and semi-dry transfer was conducted in the following order: “filter paper → gel → membrane → filter paper” (parameters were set according to the molecular weight of the target proteins). The transferred membrane was blocked at room temperature for 10 min, then washed three times with Tris-buffered saline with Tween 20 (TBST). Primary antibody was added, and the membrane was incubated overnight at 4 °C. After incubation with secondary antibody at room temperature for 2 h, the membrane was washed three times with TBST (10 min per wash), and protein bands were visualized using enhanced chemiluminescence (ECL) detection.

### Nile red analysis

FMDV-infected or drug-treated BHK-21 cells were fixed with 4% paraformaldehyde at room temperature for 15 min. Subsequently, Nile red staining solution (Beyotime, C2053S) was added, and the cells were incubated in the dark at room temperature for 20 min. DAPI staining solution (absin, abs47047616) was then added to stain cell nuclei for 10 min. Green fluorescence was observed under a confocal microscope with excitation at 485 nm.

### Data statistics

The experiments in this study were repeated at least three times using GraphPad Prism 9.5.1 software with *t*-tests and one-way analysis of variance (ANOVA). A *p*-value < 0.05 was considered a statistically significant difference (*p < 0.05; **p < 0.01; ***p < 0.001). Using ImageJ software, mean fluorescence intensity analysis and western blot gray value analysis were performed.

## Results

### Effect of the fatty acid de novo synthesis pathway on FMDV replication

#### The ACC inhibitor TOFA inhibits viral replication

**Figure 1:**
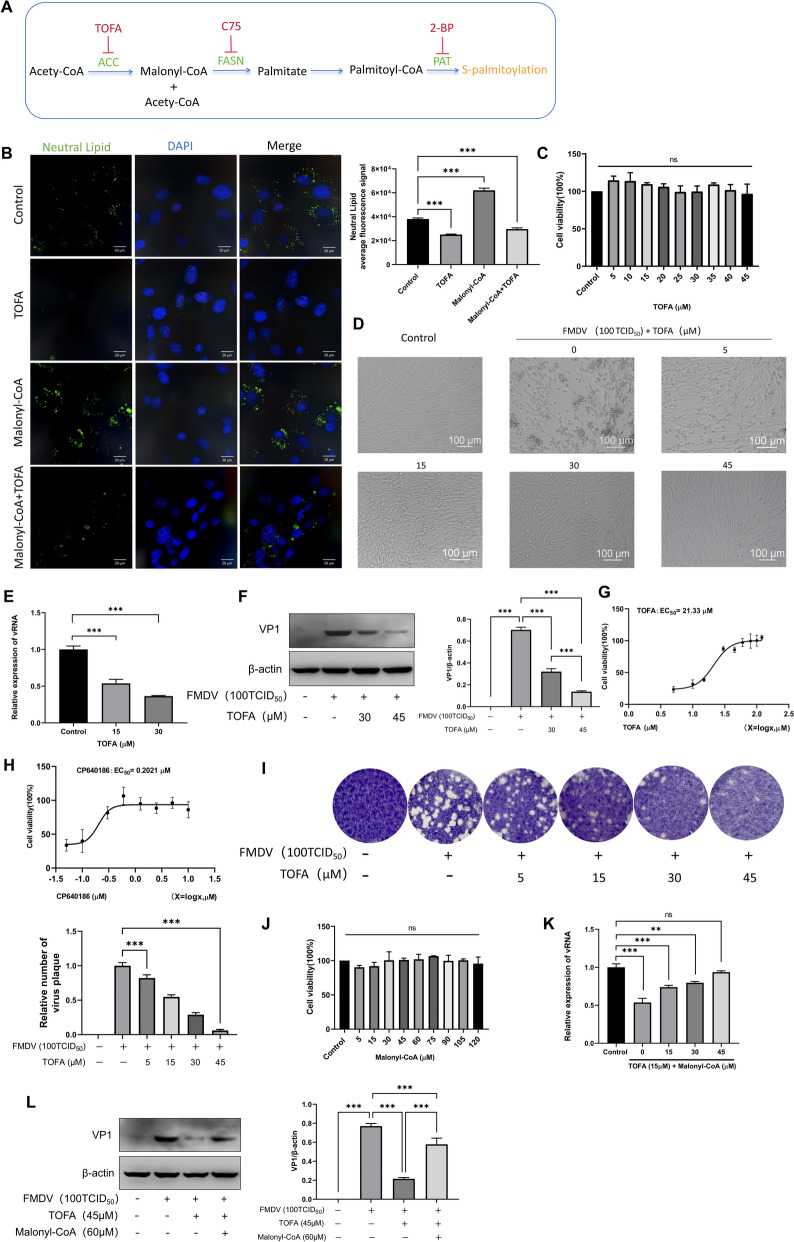
**The effect of ACC on FMDV replication.** **A** Research pathway map for de novo fatty acid synthesis. **B** BHK-21 cells were treated with 30 μM TOFA, 60 μM malonyl‑CoA, or 30 μM TOFA plus 60 μM malonyl‑CoA. After fixation, cells were stained with Nile red, and nuclei were labeled with DAPI (blue). Scale bar, 20 µm. **C** BHK-21 cells were treated with 5–45 μM TOFA for 24 h, and cell viability was not significantly affected. **D** Cytopathic effect (CPE) following FMDV infection in the presence of increasing concentrations of TOFA. **E** RT–qPCR confirmed that TOFA treatment inhibits FMDV replication. **F** Western blot analysis confirmed that TOFA treatment suppresses FMDV replication. **G** TOFA inhibited FMDV replication by 50% (EC_50_) at 21.33 μM. **H** CP640186 inhibited FMDV replication by 50% (EC_50_) at 0.2021 μM. **I** Viral plaque assay demonstrated that TOFA treatment significantly reduces FMDV replication. **J** BHK-21 cells were treated with 5–120 μM malonyl‑CoA for 24 h, and cell viability was not substantially affected. **K** RT–qPCR showed that, in the presence of 15 μM TOFA, supplementation with 0–45 μM malonyl‑CoA partially restored FMDV replication.** L** Western blot analysis confirmed that, in the presence of 45 μM TOFA, addition of 60 μM malonyl‑CoA partially rescued the expression of FMDV VP1 protein.

Acetyl-CoA carboxylase (ACC) is the first key enzyme in de novo fatty acid synthesis, which catalyzes the carboxylation of acetyl-CoA to form malonyl-CoA (Figure 1A). TOFA is an allosteric inhibitor of ACC that effectively suppresses lipid biosynthesis [10, 19]. To verify whether the de novo fatty acid synthesis pathway affects FMDV replication, BHK-21 cells were preincubated with TOFA for 12 h prior to viral infection, followed by the addition of TOFA again for a further 12 h of co-incubation with the virus, and the results were observed. Nile red fluorescent staining solution is a lipid-specific dye that stains neutral lipids with green fluorescence [20], and neutral lipids are mainly stored in LDs. Staining results showed that green fluorescence was significantly reduced after TOFA treatment, indicating that TOFA markedly inhibited lipid production (Figure 1B). Additionally, TOFA exhibited no cytotoxicity to BHK-21 cells at concentrations ranging from 5 to 45 μM (Figure 1C). Microscopic observation revealed that pretreatment of cells with TOFA prior to FMDV infection significantly alleviated the CPE in a dose-dependent manner. TOFA showed obvious antiviral viability at concentrations of 15 μM and above, and CPE was almost abolished at 30–45 μM (Figure 1D). RT–qPCR and western blot results demonstrated that TOFA treatment significantly reduced FMDV replication (Figure 1E,F). The half-maximal effective concentration (EC_50_) of TOFA for inhibiting 50% of FMDV replication was calculated to be 21.33 μM (Figure 1G). Furthermore, CP640186, another ACC inhibitor, also exhibited significant antiviral viability with an EC_50_ value of 0.2021 μM, which was much lower than that of TOFA, indicating stronger anti-FMDV potency (Figure 1H). Plaque assay further confirmed that the number of plaques was significantly reduced with increasing TOFA concentrations, and almost no plaques were formed at 45 μM (Figure 1I).

To further confirm that the inhibitory effect of TOFA on FMDV replication is attributed to the downstream reduction in lipid levels resulting from ACC inhibition, malonyl-CoA—the enzymatic product of ACC-catalyzed reactions—was supplemented in the presence of TOFA. Nile red staining results showed that supplementation with malonyl-CoA significantly increased lipid accumulation (Figure 1B). The effect of different concentrations of malonyl-CoA on BHK-21 cell viability was detected using a CCK-8 kit, and the results indicated that malonyl-CoA at concentrations ranging from 5 to 120 μM exerted no significant effect on BHK-21 cell viability (Figure 1J). RT–qPCR and western blot results demonstrated that the addition of malonyl-CoA significantly restored FMDV replication (Figure 1K,L).

Taken together, the ACC inhibitor TOFA effectively inhibits FMDV replication in BHK-21 cells, and this inhibitory effect is associated with the suppression of host de novo fatty acid synthesis. This finding suggests that targeting the host fatty acid synthesis pathway represents a potential strategy for inhibiting FMDV replication.

#### The FASN inhibitor C75 inhibits viral replication

**Figure 2:**
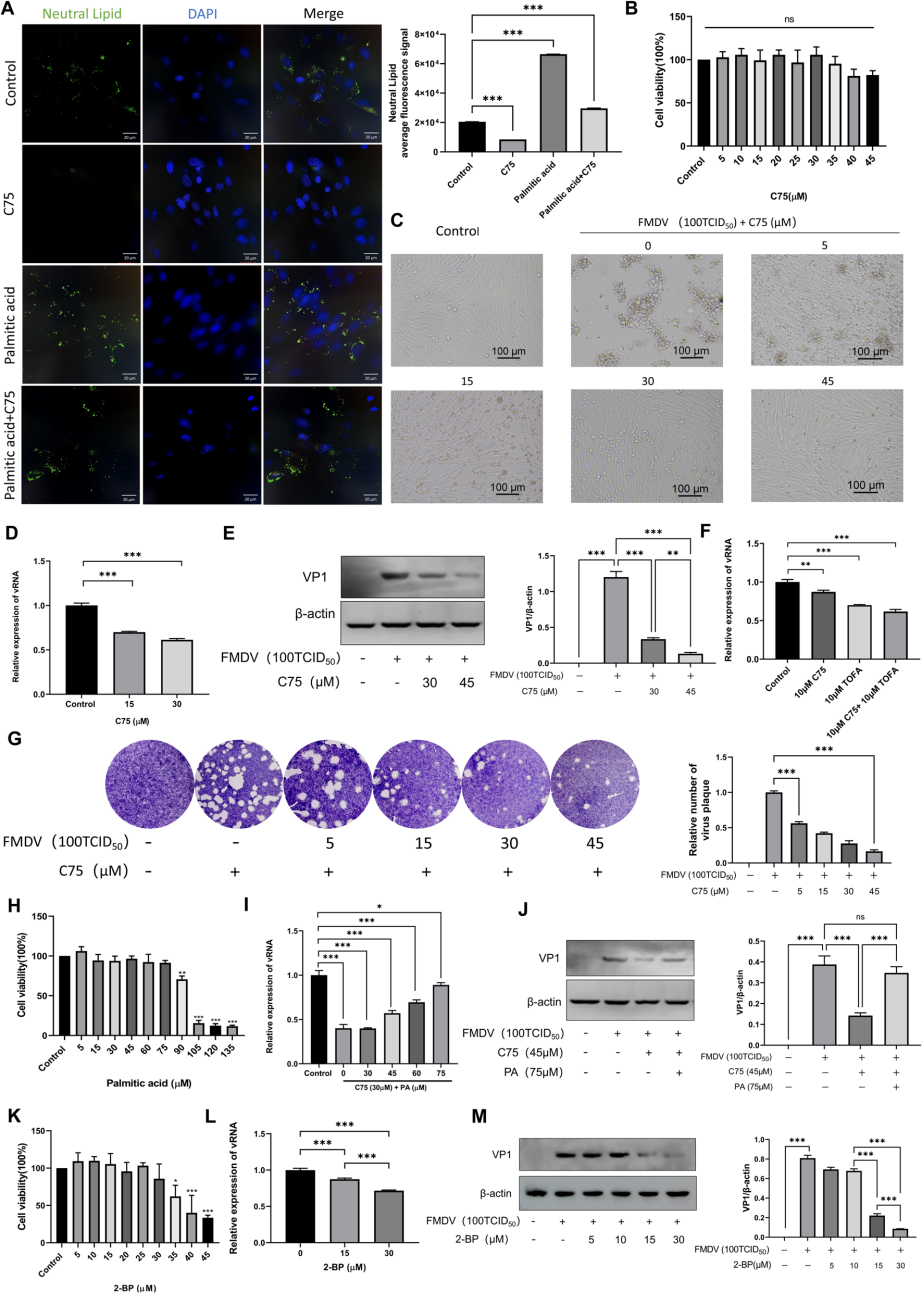
**The effect of FASN on FMDV replication.** **A** BHK-21 cells were treated with 30 μM C75, 75 μM palmitic acid, or 30 μM C75 plus 75 μM palmitic acid. After fixation, cells were stained with Nile red. Scale bar, 20 µm.** B** BHK-21 cells were treated with 5–45 μM C75 for 24 h, and cell viability was not significantly affected. **C** Cytopathic effect (CPE) following FMDV infection in the presence of increasing concentrations of C75.** D** RT–qPCR confirmed that C75 treatment inhibits FMDV replication. **E** Western blot analysis verified that C75 treatment suppresses the expression of FMDV VP1 protein. **F** Combined treatment with 10 μM C75 and TOFA exerted a stronger inhibitory effect on FMDV replication. **G** Viral plaque assay demonstrated that C75 treatment significantly inhibits FMDV replication. **H** BHK-21 cells were treated with 5–75 μM palmitic acid for 24 h, and cell viability was not substantially affected. **I** RT–qPCR showed that, in the presence of 30 μM C75, supplementation with 0–75 μM palmitic acid partially restored FMDV replication.** J** Western blot analysis confirmed that, in the presence of 45 μM C75, addition of 75 μM palmitic acid partially rescued FMDV VP1 protein expression. **K** BHK-21 cells were treated with 5–30 μM 2-bromopalmitate (2‑BP) for 24 h, and cell viability was not significantly affected. **L** RT–qPCR confirmed that 2‑BP treatment inhibits FMDV replication.** M** Western blot analysis verified that 2‑BP treatment suppresses the expression of FMDV VP1 protein.

FASN is the second key enzyme in the de novo fatty acid synthesis pathway, which catalyzes the stepwise dehydration and condensation of acetyl-CoA and malonyl-CoA to form 16-carbon palmitic acid (Figure 1A). C75 is an inhibitor of FASN that significantly suppresses FASN viability [21]. To further verify whether the de novo fatty acid synthesis pathway affects FMDV replication, BHK-21 cells were preincubated with C75 for 12 h prior to viral infection, followed by the addition of C75 again for a further 12 h of co-incubation with the virus, and the results were observed. Nile red staining results showed that C75 significantly inhibited lipid production (Figure 2A). Additionally, C75 at concentrations ranging from 5 to 45 μM exerted no significant effect on BHK-21 cell viability (Figure 2B). Microscopic observation of the antiviral effect of C75 in BHK-21 cells revealed that C75 began to exert antiviral viability at 5 μM, the antiviral effect was remarkable at 30 μM, and almost no CPE was observed at 45 μM, indicating that C75 exhibits a potent antiviral effect against FMDV (Figure 2C). RT–qPCR and western blot assays were performed to further validate the antiviral effect of C75 at the genetic and protein levels. The results demonstrated that the addition of C75 significantly reduced FMDV replication (Figure 2D,E). Furthermore, this study compared the antiviral potency of TOFA and C75. As shown in Figure 2F, when C75 and TOFA were used alone at the same concentration, TOFA exhibited a stronger inhibitory effect, and the combination of the two inhibitors achieved the optimal inhibitory effect (Figure 2F). Plaque assay further confirmed that the number of plaques formed was significantly reduced with increasing C75 concentrations, and almost no plaques were observed at 45 μM (Figure 2G).

To further confirm that the inhibitory effect of C75 on FMDV replication is attributed to the downstream reduction in lipid levels resulting from FASN inhibition, palmitic acid (PA)—the enzymatic product of FASN-catalyzed reactions—was supplemented in the presence of C75 (Figure 1A). Nile red staining results showed that PA supplementation significantly increased lipid accumulation in BHK-21 cells (Figure 2A). Prior to PA supplementation, the effect of PA on BHK-21 cell viability was evaluated using a CCK-8 kit. The results indicated that PA at concentrations ranging from 5 to 75 μM exerted no significant effect on BHK-21 cell viability, while concentrations of 90 to 135 μM impaired cell viability (Figure 2H). RT–qPCR and western blot results demonstrated that the addition of exogenous PA abrogated the inhibitory effect on FMDV replication (Figure 2I,J). Furthermore, PA can be esterified to form palmitoyl-CoA, which serves as a key substrate for palmitoylation (Figure 1A). The palmitate analog 2-bromopalmitate (2-BP) is usually used to inhibit palmitoylation [22]. Therefore, cells infected with FMDV were treated with 2-BP, and the results showed that 2-BP inhibited FMDV replication in a dose-dependent manner without reducing cell viability (Figure 2K–M). To summarize, FASN-mediated de novo fatty acid synthesis is involved in FMDV replication, and palmitoylation may be involved in it.

### Effect of lipid droplets on FMDV replication

**Figure 3:**
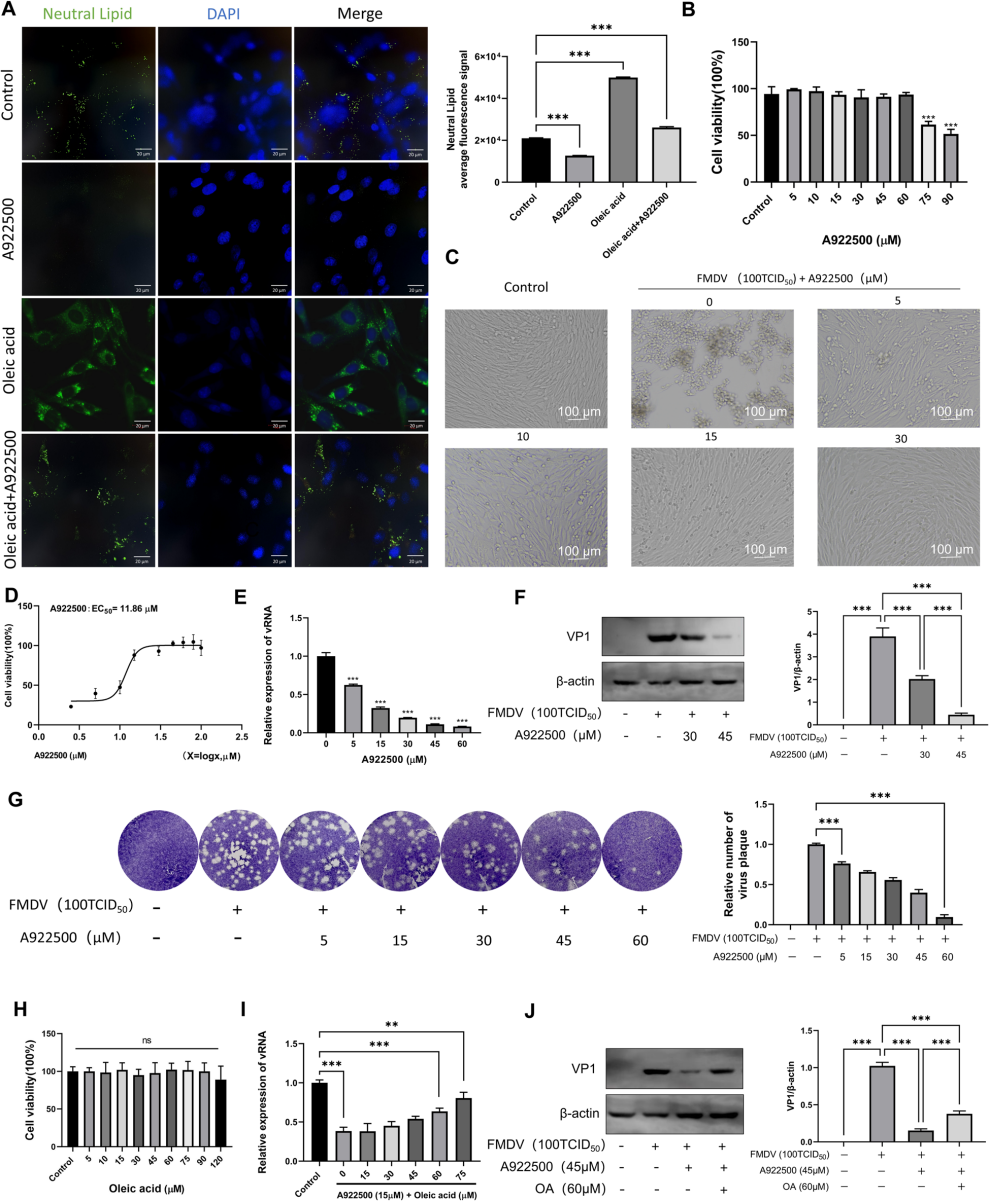
**The effect of LDs on FMDV replication.** **A** BHK-21 cells were treated with 30 μM A922500, 60 μM oleic acid, or 30 μM A922500 plus 60 μM oleic acid. After fixation, cells were stained with Nile red. Scale bar, 20 µm. **B** BHK-21 cells were treated with 5–60 μM A922500 for 24 h, and cell viability was not significantly affected. **C** Cytopathic effect (CPE) following FMDV infection in the presence of increasing concentrations of A922500.** D** A922500 inhibited FMDV replication by 50% (EC_50_) at 11.86 μM. **E** RT–qPCR confirmed that A922500 treatment inhibits FMDV replication. **F** Western blot analysis verified that A922500 treatment suppresses FMDV VP1 protein expression. **G** Viral plaque assay demonstrated that A922500 treatment significantly inhibits FMDV replication. **H** BHK-21 cells were treated with 5–120 μM oleic acid for 24 h, and cell viability was not substantially affected. **I** RT–qPCR showed that, in the presence of 15 μM A922500, supplementation with 0–75 μM oleic acid partially restored FMDV replication. **J** Western blot analysis confirmed that, in the presence of 45 μM A922500, addition of 60 μM oleic acid partially rescued FMDV VP1 protein expression.

DGAT-1 is a key enzyme in LD formation, which catalyzes the conversion of fatty acids into triglycerides (TG) for storage in LDs. It is commonly used to investigate the relationship between LDs and viral replication [23, 24]. To verify whether LDs are critical host factors for FMDV replication, BHK-21 cells were treated with the DGAT-1 inhibitor A922500. Cells were preincubated with A922500 for 12 h prior to viral infection, followed by the addition of A922500 again for a further 12 h of co-incubation with the virus, and the results were observed. Nile red staining results showed that green fluorescence was significantly reduced after A922500 treatment, indicating that A922500 markedly inhibited lipid production (Figure 3A). Additionally, A922500 exerted no significant effect on BHK-21 cell viability at concentrations ranging from 5 to 60 μM (Figure 3B). Microscopic observation of the antiviral effect of A922500 in BHK-21 cells revealed that A922500 began to exert antiviral viability at 5 μM, the antiviral effect was further enhanced at 10–15 μM, and almost no obvious CPE was observed when the concentration was increased to 30 μM (Figure 3C). The EC50 of A922500 was determined using a CCK-8 kit, and the results showed that the EC50 of A922500 was 11.86 μM, meaning that A922500 could inhibit 50% of FMDV replication at this concentration (Figure 3D). RT–qPCR and western blot assays were performed to further validate the antiviral effect of A922500 at the genetic and protein levels. The experimental results demonstrated that compared with the control group, A922500 treatment reduced FMDV replication in a dose-dependent manner (Figure 3E,F). Consistent results were obtained in the plaque assay, where the A922500-treated group significantly decreased FMDV replication (Figure 3G). Taken together, the DGAT-1 inhibitor A922500 effectively inhibits FMDV replication in BHK-21 cells, and LDs are critical host factors for FMDV replication.

Oleic acid (OA), a monounsaturated long-chain fatty acid, is widely distributed in nature and one of the most common unsaturated fatty acids. When cells take up excess fatty acids (e.g., OA), the fatty acids are converted into neutral lipids and stored in LDs, thereby avoiding the potential toxicity of FFAs. To further verify the relationship between LD formation and FMDV replication, OA was added to the culture medium containing A922500 to induce LD biogenesis. Nile red staining results showed that OA supplementation significantly increased intracellular lipid accumulation (Figure 3A). To rule out the interference of OA-induced reduced cell viability on the experimental results, BHK-21 cells were co-incubated with OA at concentrations ranging from 5 to 120 μM for 24 h, and cell viability was then detected. The results indicated that OA exerted no significant effect on BHK-21 cell viability at concentrations of 5 to 120 μM (Figure 3H). RT–qPCR and western blot assays were performed to validate the effect at the genetic and protein levels. The results demonstrated that compared with the control group, the inhibitory effect of A922500 was gradually attenuated with increasing OA concentrations (Figure 3I,J).

### Effects of CPT1A on FMDV replication

**Figure 4:**
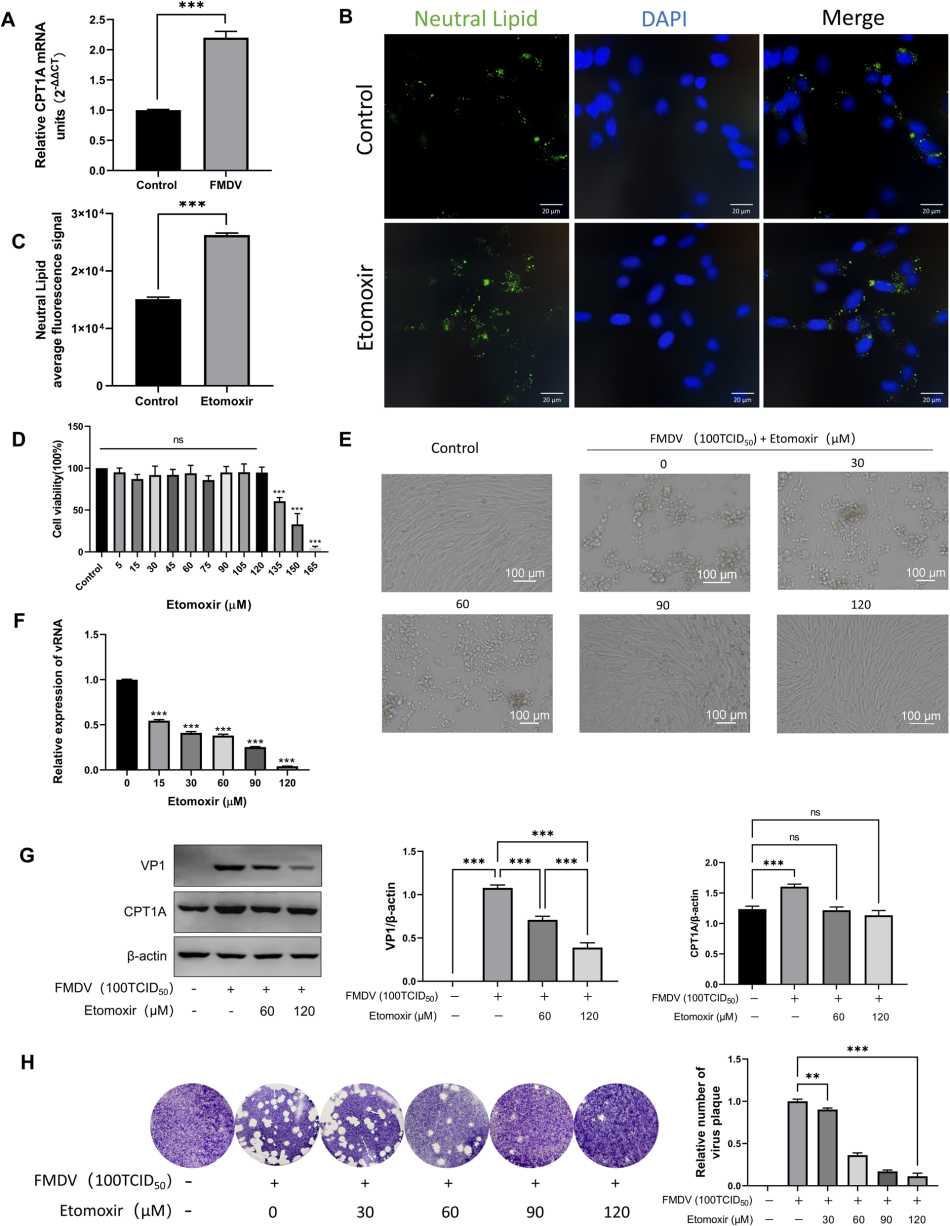
**The effect of CPT1A on FMDV replication. A** The expression of CPT1A was significantly upregulated after FMDV infection. **B**, **C** BHK-21 cells were treated with 60 μM Etomoxir. After fixation, cells were stained with Nile red, and nuclei were labeled with DAPI (blue). Scale bar, 20 µm. **D** BHK-21 cells were treated with 5–120 μM Etomoxir for 24 h, and cell viability was not significantly affected. **E** Cytopathic effect (CPE) following FMDV infection in the presence of increasing concentrations of Etomoxir. **F** RT–qPCR confirmed that Etomoxir treatment inhibits FMDV replication. **G** Western blot analysis verified that Etomoxir treatment suppresses FMDV replication. **H** Viral plaque assay demonstrated that Etomoxir treatment significantly inhibits FMDV replication.

Carnitine palmitoyltransferase 1A (CPT1A) is a mitochondrial enzyme that catalyzes the conjugation of long-chain fatty acids with carnitine to form acylcarnitines, which enables them to cross the inner mitochondrial membrane and enter the mitochondrial matrix for β-oxidation. As the rate-limiting step of fatty acid oxidation, CPT1A plays a crucial role in lipid metabolism [25]. In this study, we found that the messenger RNA (mRNA) and protein expression levels of CPT1A were significantly upregulated in FMDV-infected cells, suggesting that CPT1A may serve as a host factor for FMDV replication (Figure 4A,G). To further explore the relationship between CPT1A and FMDV replication, we used Etomoxir, an irreversible inhibitor of CPT1A that prevents long-chain fatty acids from entering mitochondria for β-oxidation [25, 26]. Nile red staining results showed that Etomoxir treatment significantly increased intracellular lipid accumulation, indicating that Etomoxir successfully blocked fatty acid entry into mitochondria for β-oxidation (Figure 4B,C). The cytotoxicity of Etomoxir was evaluated using a CCK-8 kit, and the results demonstrated that Etomoxir exerted no significant effect on BHK-21 cell viability at concentrations ranging from 5 to 120 μM (Figure 4D). Microscopic observation of the antiviral effect of Etomoxir revealed that almost no obvious CPE was observed at 120 μM, indicating that Etomoxir possesses potent antiviral viability against FMDV (Figure 4E). RT–qPCR and western blot assays were performed to further validate the antiviral effect of Etomoxir at the mRNA and protein levels. The results showed that Etomoxir treatment significantly inhibited FMDV replication, and the inhibitory effect was more pronounced with increasing Etomoxir concentrations (Figure 4F,G). Consistent results were obtained in the plaque assay, which further supports the critical role of CPT1A in FMDV replication (Figure 4H). Taken together, the CPT1A inhibitor Etomoxir effectively inhibits FMDV replication in BHK-21 cells.

## Discussion

FMDV replication is closely associated with lipid metabolism, and the de novo fatty acid synthesis pathway is a critical component of lipid metabolic pathways. The activities of its two key enzymes, ACC and FASN, are essential for the synthesis of cell membranes and various lipid substrates. Currently, these enzymes have been widely used as therapeutic targets in RNA virus research [11, 12, 27, 28]. In this study, we used TOFA, C75, and 2-BP—inhibitors of ACC, FASN, and fatty acid palmitoylation, respectively—to verify the role of the de novo fatty acid synthesis pathway in FMDV replication. The results demonstrated that these three inhibitors significantly suppressed FMDV replication, a finding consistent with previous studies on classical CSFV, which also possesses a single-stranded positive-sense RNA genome [11]. Notably, supplementation with their respective enzymatic products (malonyl-CoA for TOFA and palmitic acid for C75) in the culture medium containing TOFA or C75 abrogated the viral inhibition. This is consistent with earlier research showing that malonyl-CoA and palmitic acid can restore CSFV replication that was inhibited by these inhibitors [11]. Collectively, these results preliminarily indicate that, similar to other RNA viruses, FMDV replication relies on the host cell’s de novo fatty acid synthesis pathway.

LDs are critical organelles for neutral lipid storage and central hubs of lipid metabolism in cells. They not only participate in multiple cellular processes, such as lipid homeostasis, membrane trafficking, and signal transduction [9], but also play an important role in viral replication [29], for instance, serving as an energy source for viral replication. Following DENV infection, autophagosome formation is induced to promote LD breakdown and release FFAs, which then enter mitochondria to generate ATP that fuels DENV replication [30]. Studies on severe acute respiratory syndrome coronavirus 2 (SARS-CoV-2) have shown that treatment with A922500, an inhibitor of DGAT-1 (a key enzyme in LD biogenesis), significantly reduces SARS-CoV-2 replication [24], indicating that LDs are critical host factors for SARS-CoV-2 replication. In the present study, we similarly treated FMDV-infected cells with A922500 to investigate the role of LDs in FMDV replication. The results demonstrated that A922500 significantly inhibited FMDV replication in a dose-dependent manner, which is consistent with the findings from SARS-CoV-2 studies. OA, a common monounsaturated fatty acid, can induce LD biogenesis. Supplementation with OA in the culture medium containing A922500 increased LD accumulation and restored FMDV replication, a result also confirmed in studies on ZIKV [31]. Furthermore, reports have shown that the FMDV 2C protein co-localizes with LDs in FMDV replication complexes, and overexpression of the 2C protein leads to the accumulation of LDs around the nucleus [16], suggesting that LDs may be regulated by the 2C protein to participate in FMDV replication. Collectively, these results indicate that LDs, as central hubs of lipid metabolism, are involved in FMDV replication.

Viral genome replication, protein synthesis, and virion assembly require substantial energy, and the fatty acid β-oxidation pathway can provide energy for viral replication. Studies have shown that DENV [30], EVA-71 [10], and BoHV-1 [32]—which, similar to FMDV, are RNA viruses—fuel their replication via the fatty acid β-oxidation pathway. Among these, EVA-71 upregulates CPT1A protein expression to accelerate the transport rate of fatty acids into mitochondria, thereby providing energy for its replication [10]. In the present study, we observed a similar phenomenon: The mRNA and protein expression levels of CPT1A were significantly upregulated following FMDV infection, suggesting that CPT1A may serve as a critical host factor for FMDV replication. To further confirm whether CPT1A plays a role in FMDV replication, we investigated the effect of Etomoxir (a CPT1A inhibitor) on FMDV replication by treating cells with this compound. The results showed that Etomoxir inhibited FMDV replication in a dose-dependent manner, which is consistent with previous reports demonstrating that Etomoxir suppresses the replication of EVA-71 [10] and BoHV-1 [32]. Collectively, these findings indicate that CPT1A is a critical host factor for FMDV replication.

**Figure 5:**
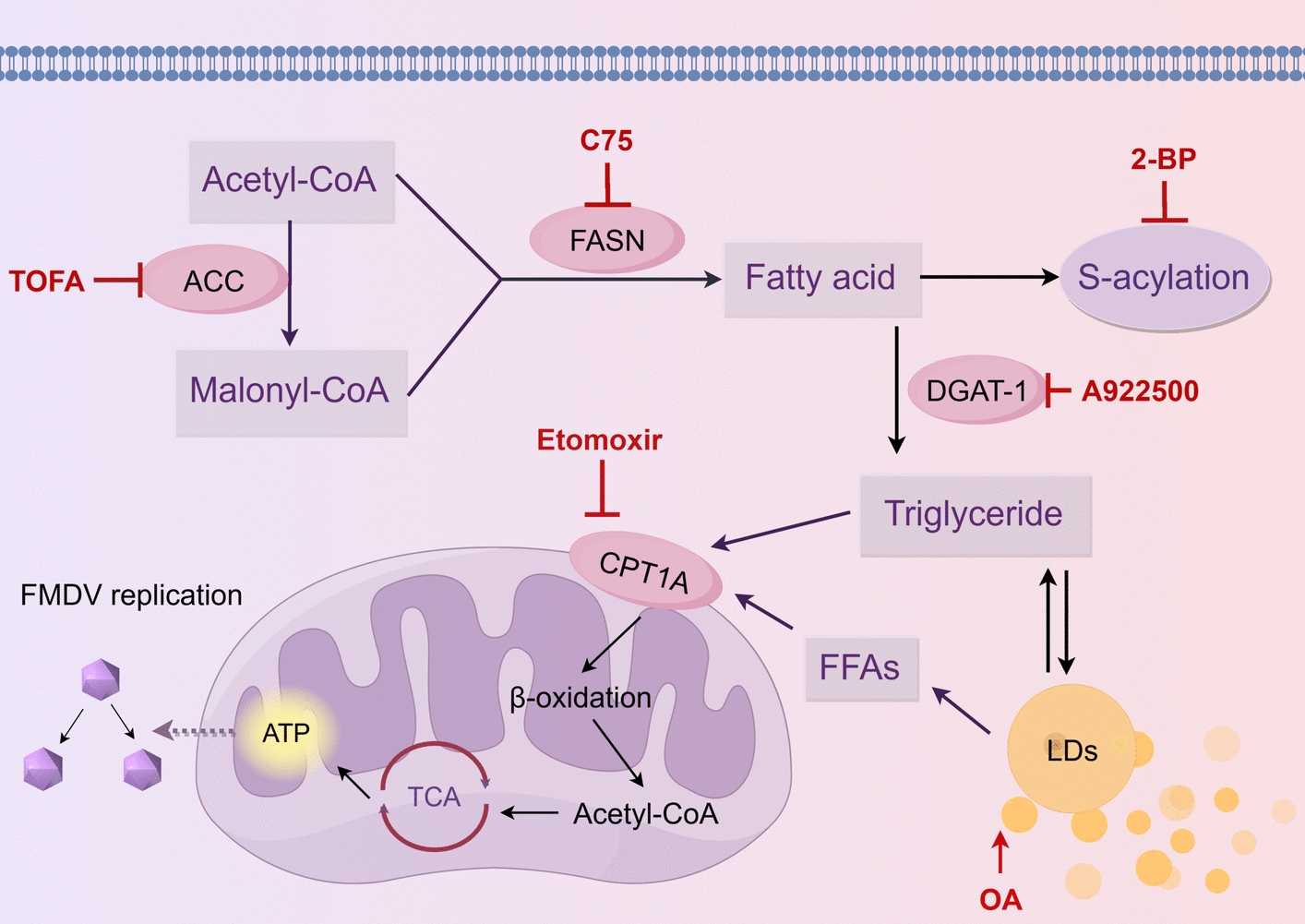
**Utilization of the lipid metabolism model during FMDV infection.** ACC catalyzes the carboxylation of acetyl-CoA to generate malonyl-CoA; malonyl-CoA and acetyl-CoA are catalyzed by FASN to produce fatty acids; fatty acids are catalyzed by DGAT-1 to form triglycerides, which are stored in LDs; the degradation of LDs generates a large amount of FFAs, which are transported into mitochondria under the action of CPT1A to participate in β-oxidation for ATP production.

In summary, data from the present study demonstrate that ACC and FASN—key enzymes in the de novo fatty acid synthesis pathway DGAT-1, a key enzyme in LD biogenesis, and carnitine CPT1A, a key enzyme in fatty acid β-oxidation, are critical host factors for FMDV replication. These findings are summarized in a working model (Figure 5). Inhibition of these two key enzymes in the fatty acid synthesis pathway impairs FMDV replication, indicating that FMDV replication is dependent on the de novo fatty acid synthesis pathway. The fatty acid synthesis pathway generates numerous long-chain fatty acids; among these, palmitic acid is esterified to form palmitoyl-CoA, which serves as a substrate for palmitoylation and may be involved in the palmitoylation modification of FMDV proteins. Other fatty acids are catalyzed by DGAT-1 and other enzymes to form triglycerides, which are stored in LDs. FMDV infection upregulates CPT1A expression, facilitating fatty acid entry into mitochondria, which may provide energy for FMDV replication.

## Data Availability

All data underlying the results are available in the article, and no additional source data are needed.
